# Intravascular *Schistosoma mansoni* Cleave the Host Immune and Hemostatic Signaling Molecule Sphingosine-1-Phosphate *via* Tegumental Alkaline Phosphatase

**DOI:** 10.3389/fimmu.2018.01746

**Published:** 2018-07-30

**Authors:** Manal Elzoheiry, Akram A. Da’dara, Rita Bhardwaj, Qiang Wang, Manar S. Azab, El-Saeed I. El-Kholy, Samar N. El-Beshbishi, Patrick J. Skelly

**Affiliations:** ^1^Molecular Helminthology Laboratory, Department of Infectious Disease and Global Health, Cummings School of Veterinary Medicine, Tufts University, North Grafton, MA, United States; ^2^Department of Medical Parasitology, Faculty of Medicine, Mansoura University, Mansoura, Egypt

**Keywords:** sphingosine-1-phosphate, host–parasite interaction, schistosome, *Schistosoma mansoni* alkaline phosphatase, alkaline phosphatase

## Abstract

Schistosomes are parasitic flatworms that infect the vasculature of >200 million people around the world. These long-lived parasites do not appear to provoke blood clot formation or obvious inflammation around them *in vivo*. Proteins expressed at the host–parasite interface (such as *Schistosoma mansoni* alkaline phosphatase, SmAP) are likely key to these abilities. SmAP is a glycoprotein that hydrolyses the artificial substrate *p*-nitrophenyl phosphate in a reaction that requires Mg^2+^ and at an optimal pH of 9. SmAP additionally cleaves the nucleoside monophosphates AMP, CMP, GMP, and TMP, all with a similar Km (~600–650 μM). Living adult worms, incubated in murine plasma for 1 h, alter the plasma metabolome; a decrease in sphingosine-1-phosphate (S1P) is accompanied by an increase in the levels of its component parts—sphingosine and phosphate. To test the hypothesis that schistosomes can hydrolyze S1P (and not merely recruit or activate a host plasma enzyme with this function), living intravascular life-stage parasites were incubated with commercially obtained S1P and cleavage of S1P was detected. Parasites whose SmAP gene was suppressed using RNAi were impaired in their ability to cleave S1P compared to controls. In addition, recombinant SmAP hydrolyzed S1P. Since extracellular S1P plays key roles in controlling inflammation and platelet aggregation, we hypothesize that schistosome SmAP, by degrading S1P, can regulate the level of this bioactive lipid in the environment of the parasites to control these processes in the worm’s local environment. This is the first report of any parasite being able to cleave S1P.

## Introduction

Schistosomiasis is a parasitic disease caused by platyhelminths of the genus *Schistosoma*. More than 200 million people are infected with these worms globally, with >800 million living at risk of infection (1, 2). There are three major species that infect humans; these are *Schistosoma mansoni, S. japonicum*, and *S. haematobium*. Infection occurs when cercariae (larval forms) emerge from an intermediate freshwater snail host and enter the skin of the final human host. Here, they transform into juvenile forms called schistosomula. Schistosomula migrate beneath the dermis to locate and enter a blood vessel. Once in the circulation, the young worms circulate to the liver. Here, they mature into adult males and females, mate, and migrate to the blood vessels around the intestines or the bladder (depending on the species) where egg laying commences. Disease arises largely as a result of the host’s inflammatory immune response to parasite eggs that do not exit the body but get trapped internally. The result is local and systemic pathological effects including anemia, growth stunting and impaired cognition, as well as organ-specific effects like hepatosplenomegaly, periportal fibrosis with portal hypertension, and urogenital inflammation.

Schistosomes possess mechanisms to avoid immune elimination and so can live for many years in the human blood stream (3, 4). Furthermore, the parasites appear not to provoke blood clot formation around them within the vasculature (5). Thus, the relatively large adult worms can be found in the bloodstream mostly unmolested by elements of the host’s immune and coagulation systems. Host-interactive proteins found in the parasite’s tegument (skin) have been hypothesized to be critical to the worm’s ability to dampen host immunity as well as to hamper thrombus formation (6, 7). For example, the worms express an adenosine triphosphate (ATP) diphosphohydrolase—SmATPDase1 (8, 9), detected in the adult tegument by immunolocalization (10, 11), which can cleave the proinflammatory mediator ATP (12). SmATPDase1 can also cleave adenosine diphosphate (ADP)—a potently pro-thrombotic molecule (12). The worms additionally possess an ectonucleotide pyrophosphatase/phosphodiesterase designated SmNPP5 that can cleave ADP (13). Like SmATPDase1, SmNPP5 is found in the adult tegument by immunolocalization (14, 15) and has been demonstrated to impede platelet aggregation *in vitro* (13). The genes encoding both SmNPP5 and SmATPDase1 are quickly turned on in the parasite’s blood stages, following invasion of the final host and the proteins are highly expressed in the host-interactive tegumental membranes (12, 14, 15). In this location, it is likely that these ecto-enzymes encounter and hydrolyze host proinflammatory substrates (like ATP) as well as pro-thrombotic substrates (like ADP). Controlling local ATP and ADP levels may help schistosomes to impede inflammation and blood clotting in their local environment and so prolong worm survival.

In this study, we focus on an additional *S. mansoni* enzyme—alkaline phosphatase (SmAP). This ~60 kDa, GPI-anchored protein is highly expressed in intravascular parasite life stages and localizes both at the parasite surface as well as internally (16, 17). It has been shown that live schistosomes can produce adenosine using exogenous AMP. This ability is effectively eliminated following SmAP gene suppression using RNAi (16). This result suggests that tegumental SmAP accesses exogenous AMP and cleaves it, thus generating adenosine. Extracellular adenosine is a powerful immunosuppressant that can dampen host immune responses (18–20) and can inhibit platelet activation and thrombus formation (21). Thus, SmAP (like SmATPDase1 and SmNPP5) may act to skew the immediate biochemical environment of schistosomes in an anti-inflammatory and antithrombotic direction.

In this work, we set out to express recombinant SmAP (rSmAP) and characterize the active enzyme; of particular interest is the identification of other host signaling molecules upon which SmAP may impinge. In this regard, we find that all intravascular life stages, as well as SmAP, can cleave sphingosine-1-phosphate (S1P). This compound is a bioactive metabolite of sphingolipid metabolism that influences a wide range of normal physiological functions, including cell survival, proliferation, migration, and differentiation (22, 23). S1P triggers a family of G-protein-coupled receptors to initiate signaling pathways that drive these processes (24). In the extracellular environment, S1P impacts vascular permeability, inflammation, and platelet aggregation (25–29). S1P signaling regulates key aspects of immune cell biology (25, 26, 30) including the trafficking of lymphocytes, dendritic cells, mast cells, monocytes/macrophages, and neutrophils; S1P can also affect immune cell degranulation and inflammatory mediator production (25, 31). By promoting innate lymphoid cell migration, S1P-mediated chemotaxis has been shown to be important in antihelminth defense (32). S1P signaling is additionally coupled with coagulation processes (33, 34). The molecule has been reported to regulate platelet function by inducing platelet shape change and aggregation (35, 36). This is the first report of any parasite possessing the ability to degrade this key signaling molecule. Regulating local levels of S1P by intravascular schistosomes using SmAP may help disrupt host immune and coagulation signaling pathways and so promote parasite survival.

## Materials and Methods

### Parasites and Mice

*Biomphalaria glabrata* snails (strain NMRI), infected with *S. mansoni* were obtained from the Schistosomiasis Resource Center, Biomedical Research Institute (BRI), Rockville MD, USA. Cercariae (strain NMRI) were obtained from these snails and schistosomula were prepared as described (37). Female Swiss Webster mice were infected with ~100 *S. mansoni* cercariae and, about 6 weeks later, adult male and female parasites were recovered by perfusion. All parasites were cultured in complete DMEM/F12 medium containing 10% heat-inactivated fetal bovine serum, 200 µg/ml streptomycin, 200 U/ml penicillin, 1 µM serotonin, 0.2 µM Triiodo-l-thyronine, 8 µg/ml human insulin and were maintained at 37°C, in an atmosphere of 5% CO_2_ (38). All animal protocols were approved by the Institutional Animal Care and Use Committees (IACUC) of Tufts University under protocol G2015-113. All experimental procedures followed the approved guidelines of the IACUC.

### Expression and Purification of rSmAP

The coding sequence of SmAP (accession number, EU040139), including the signal peptide and GPI anchoring domain, was synthesized commercially (Genscript USA Inc., Piscataway, NJ, USA) in codon optimized form (using hamster codon preferences) Next, the region encoding amino acids K^29^-R^512^ (i.e., lacking the amino terminal signal peptide and the carboxyl terminal GPI anchoring signal) was produced by PCR using the synthetic SmAP DNA and forward and reverse primers containing *AscI* and *XhoI* restriction sites, respectively. The purified PCR product was cloned into the pSecTag2A plasmid (ThermoFisher Scientific) at the *Asc*I and *Xho*I sites in frame (at the 5′-end) with the Igκ leader sequence and (at the 3′-end) a myc tag and 6-histidine tag. Sequencing at the Tufts University Core Facility confirmed successful in-frame cloning.

To express rSmAP, suspension-adapted FreeStyle Chinese Hamster Ovary Cells (CHO-S cells, Invitrogen) were first grown in FreeStyle CHO Expression Medium containing 8 mM l-glutamine (Thermo Fisher Scientific) at 37°C and 8% CO_2_ with shaking. Next, cells were transfected using Free-Style Max Reagent following the manufacturer’s instructions (ThermoFisher Scientific). Transfected cells were grown as before and aliquots were recovered at different time points post-transfection to monitor viability (by Trypan Blue exclusion) and rSmAP expression (by western blotting). Optimal recombinant protein expression coupled with high cell viability was found 48–72 h after transfection.

Recombinant SmAP was recovered from 72-h cell culture medium by immobilized metal affinity chromatography (IMAC) using HisTrap™ Excel columns, according to the manufacturer’s instructions (GE Healthcare Life Sciences). Column fractions were evaluated by SDS-PAGE. Purified recombinant protein, eluted from the column, was dialyzed twice overnight at 4°C against 50 mM Tris–HCl (pH 7.4), 150 mM NaCl. The protein was then concentrated using ultrafiltration centrifugation (Pierce Protein Concentrators, 10K MWCO, ThermoFisher Scientific). A BCA Protein Assay Kit (Pierce) was used to determine the final protein concentration and an aliquot was resolved by SDS-PAGE and Biosafe Coomassie staining (BioRad) to assess purity. Western blot analysis was used (as described in the following section) to characterize rSmAP, using a commercially prepared anti-SmAP antibody (1:500) (16) or an anti-myc HRP-conjugated antibody (1:5,000; ThermoFisher Scientific).

### Deglycosylation of SmAP

Peptide-*N*-Glycosidase F (PNGase F, New England Biolabs) was employed to *N*-deglycosylate recombinant and native SmAP proteins following the manufacturer’s instructions. In brief, 5 µg of rSmAP, or 50 µg of adult male worm lysate (generated by homogenizing adult worms in ice cold PBS with protease inhibitors), was denatured at 100°C for 10 min in the presence of 40 mM DTT and 0.5% SDS. Next, NP-40 was added to 1%. PNGase F and deglycosylation buffer were added and the mixture incubated at 37°C for 3 h. Following this, the PNGase F-treated, and untreated control protein samples were resolved by 4–20% SDS-PAGE (BioRad), transferred to PVDF membrane and probed by standard western blotting using an affinity purified, rabbit anti-SmAP antibody (16). In brief, the membrane was blocked with Tris-buffered saline, pH 7.5, 0.05% Tween 20 (TBST) with 5% dry, non-fat milk powder for 1 h at ambient temperature. The membrane was next incubated with anti-SmAP antibody (1:500) for 1 h at ambient temperature, washed with TBST buffer for 30 min, and incubated with horseradish peroxidase-labeled donkey anti-rabbit IgG (1:5,000) (GE Healthcare, NJ, USA) for 1 h at ambient temperature. Signals were monitored using ECL Western Blotting Detection Reagents (GE Healthcare) and a ChemiDoc Touch Imaging system (BioRad).

### SmAP Activity Assay

To measure SmAP activity in living parasites, approximately 1,000 schistosomula or individual adult male or female worms (in replicate) were incubated in assay buffer [50 mM Tris–HCl (pH 9), 5 mM KCl, 135 mM NaCl, 10 mM glucose, 10 mM MgCl_2_] containing the substrate *p*-nitrophenyl phosphate (*p*-NPP, routinely at 2 mM) or nucleoside monophosphate (AMP, CMP, GMP, TMP, 0–2 mM) or sphingosine 1 phosphate (S1P, 0.5 mM) (16). S1P, obtained from Sigma-Aldrich, was prepared in methanol at 2.6 mM solution as recommended by the manufacturer. In some preparations, S1P was dissolved in 95% methanol, dried under nitrogen gas, and reconstituted in fatty acid-free bovine serum albumin (4 mg/ml). rSmAP was used at 0.5–5 μg/assay, as indicated, and with 0.5 mM S1P. To monitor *p*-nitrophenol generated following *p*-NPP substrate cleavage, changes in optical density at 405 nm over time were measured with a Synergy HT spectrophotometer (Bio-Tek Instruments, Winooski, VT, USA). Phosphate generated following substrate (nucleoside monophosphate or S1P) cleavage was measured using a Phosphate Colorimetric Assay Kit (BioVision), following the instructions of the manufacturer. Samples were recovered at selected time points from each reaction and substrate cleavage (phosphate generation) was monitored.

### Biochemical Characterization of rSmAP

To measure the pH preference of rSmAP, cleavage of *p*-NPP was determined over a pH range from 5.5 to 12 in the following buffers: MES (pH 5–6.5), MOPS (pH 6.5–7.5), HEPES (pH 7.0–8.0), Tris–HCl (pH 7.5–9.0), Trizma (pH 9.0), glycine-NaOH (pH 9.0–12) buffers. The 200 µl reaction mixture contained 50 mM of the appropriate buffer, 10 mM MgCl_2_, 0.5 µg rSmAP, and 2 mM *p*-NPP.

To determine the need of rSmAP for divalent ions, the standard *p*-NPP cleavage assay was carried out as described above but with the 50 mM Tris–HCl (pH 9) buffer modified to contain different concentration of MgCl_2_, CaCl_2_, ZnCl_2_, or CuCl_2_ as indicated, or 5 mM ethylenediaminetetraacetic acid (EDTA). The Michaelis–Menten equation was applied to measure the enzyme’s Michaelis constant (Km) for selected substrates. Data were analyzed and plotted using GraphPad Prism 5.0.

### Treatment of Parasites With siRNAs

Parasites were electroporated with 10 µg of either a synthetic siRNA targeting SmAP (SmAPsiRNA 1: 5′-AAGAAATCAGCAGATGAGAGATTTAAT-3′) or with a control siRNA targeting no sequence in the schistosome genome (Control: 5′-CTTCCTCTCTTTCTCTCCCTTGTGA-3′) following a protocol that yields robust suppression of SmAP gene expression (16).

### S1P, Sphingosine and Phosphate Detection

Blood was recovered from the tail veins of 10 mice into heparinized tubes. Blood cells were pelleted by brief centrifugation, and the plasma generated was pooled and aliquoted. Adult schistosomes (~50 pairs) were incubated in one 500 µl murine plasma aliquot, which was incubated at 37°C. A control aliquot (without worms) was similarly treated. Samples, collected at baseline (0 min) and after 20 and 60 min incubation with or without parasites, were subjected to metabolomic analysis at Metabolon Inc. The relative levels of three metabolites [S1P, sphingosine, and phosphate] are described, and these are extracted from a global metabolomics analysis carried out using the pipeline developed by Metabolon. At least four samples per treatment/time point were tested. Briefly, each plasma sample was prepared by solvent extraction, and the resulting extract was applied to gas chromatography/mass spectrometry (GC/MS) and liquid chromatography tandem MS (LC/MS/MS) platforms (39). S1P, sphingosine, and phosphate were each identified by their retention time and mass by comparison to purified standards. Results are expressed relative to the baseline (0 min) measurement, set at 1.

### Statistical Analysis

Data are presented as mean ± SD. Means were compared by: *t*-test (two-tailed, unpaired) for comparison of two groups; one-way ANOVA for comparison of more than two groups; repeated-measures ANOVA for comparison of more than two matched groups; and two-way ANOVA for comparison of different groups with different factors followed by a *post hoc* Bonferroni multiple comparison test using GraphPad Prism, v. 5 (GraphPad Software, Inc., San Diego, CA, USA). For metabolite comparisons, Welch’s two-sample *t*-test was used to identify biomolecules differing significantly between groups. *p* < 0.05 was considered significant.

## Results

### Expression of rSmAP

**Figure 1:**
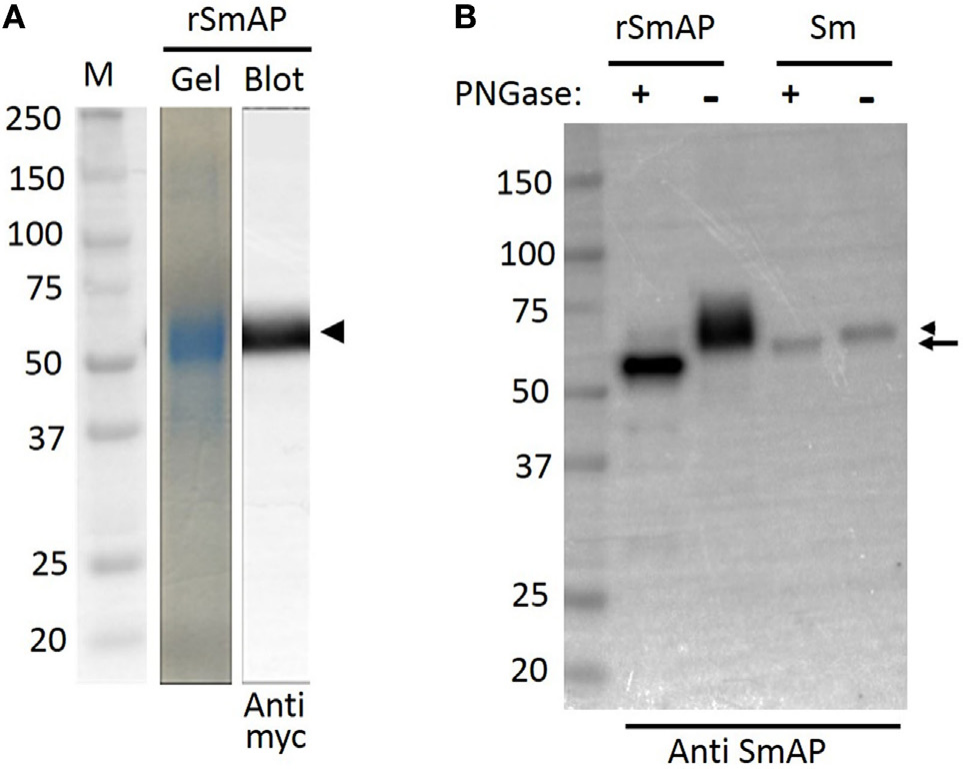
Analysis of *Schistosoma mansoni* alkaline phosphatase (SmAP) by SDS-PAGE and Western Blotting. **(A)** Following immobilized metal affinity chromatography, an aliquot of purified recombinant SmAP (rSmAP) was resolved by SDS-PAGE and the gel was stained with Coomassie Blue. A prominent band at ~60 kDa is seen (gel) that can also be detected by western blotting using an anti-myc tag antibody (blot), as indicated by the arrowhead. *M* indicates molecular size markers and the numbers given represent kDa. **(B)** Western blot analysis of SmAP glycosylation status. rSmAP or adult worm lysate (Sm) was resolved by SDS-PAGE following treatment with PNGase F (+) or after no treatment (−), as indicated. The native protein resolves as a prominent band at ~60 kDa (lane Sm −, right arrowhead). Following incubation of the worm extract with PNGase F, this larger band disappears and a slightly smaller band (at ~57 kDa) appears (lane Sm +, arrow). Incubation of rSmAP with PNGase F also leads to a notable shift in its migration profile; the broad band at ~60 kDa in the rSmAP “−” lane disappears and a band running at ~55 kDa is now seen (rSmAP+). Numbers represent kDa of molecular size markers (left lane).

Plasmid encoding a His-tagged, secreted form of SmAP was transfected into CHO-S cells and 72 h later, the culture supernatant was collected; rSmAP was recovered using standard IMAC. Figure 1A (“Gel” lane) shows purified rSmAP, eluted from the chromatography column. This protein is also detected by western blotting using an anti-myc tag antibody (Figure 1A, “Blot” lane). The arrowhead indicates rSmAP, running at ~60 kDa.

Figure 1B compares rSmAP with the native schistosome protein by western blot analysis before (−) and after (+) treatment with PNGase F. The native protein resolves as a prominent band at ~60 kDa in extracts of adult male worms (Figure 1B, lane Sm −, arrowhead). After incubating the worm extract with PNGase F, this larger band disappears and a new, slightly smaller band appears (at ~57 kDa; Figure 1B, lane “Sm +” arrow). Incubating purified rSmAP with PNGase F similarly leads to a detectable shift in its migration profile; the broad band at ~60 kDa in the rSmAP (−) lane disappears and a band running at ~55 kDa is now seen (rSmAP+). These data show that both the native and recombinant protein are glycosylated.

### Characterization of SmAP

**Figure 2:**
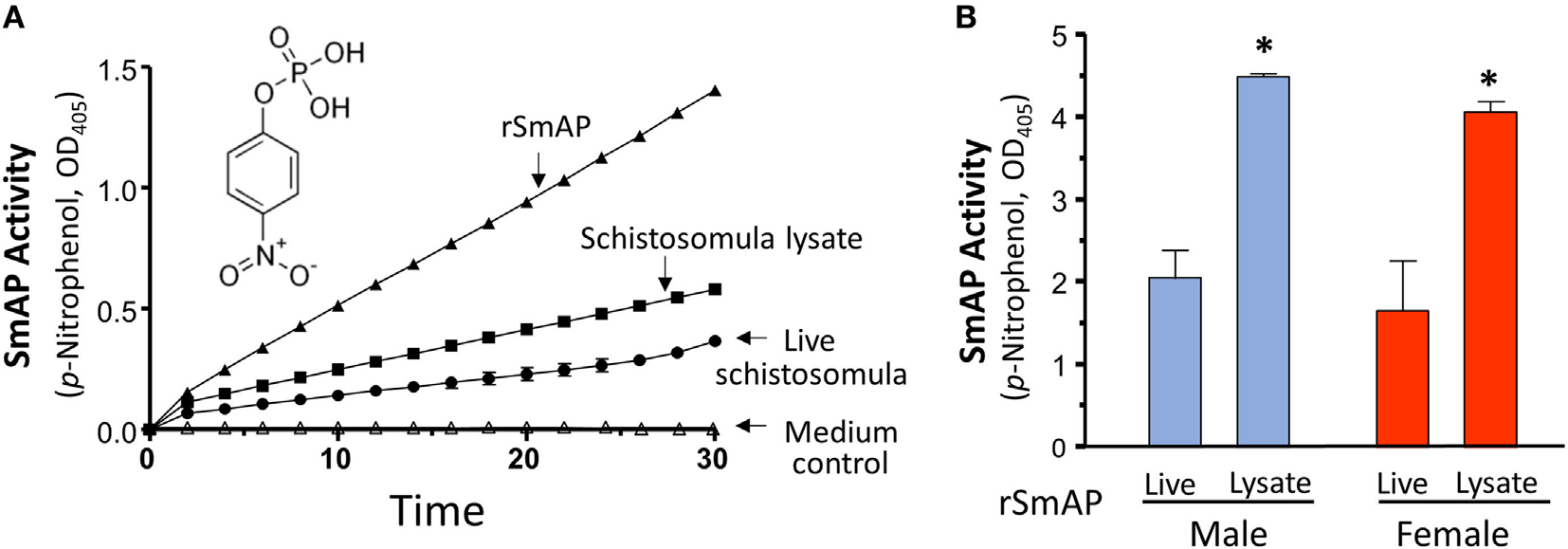
**(A)** *Schistosoma mansoni* alkaline phosphatase (SmAP) activity (*p*-NPP cleavage, mean OD_405_ ± SD) exhibited by recombinant protein (rSmAP, filled triangles) or live schistosomula (groups of 1,000, circles) or total lysates of equivalent numbers of schistosomula (squares). Medium in which schistosomula had been incubated for 24 h displayed essentially no activity (open triangles, “Medium control”). The chemical structure of SmAP substrate para-nitrophenyl phosphate (*p*-NPP) is illustrated. **(B)** SmAP activity (*p*-NPP cleavage/h, mean OD_405_ ± SD) observed in individual live adult males (blue) or females (red) compared to that detected in total lysates of individual males or females. *N* ≥ 10 in each case. * indicates that lysates of male or female worms exhibit significantly greater substrate cleavage than live male or female worms; *t*-test, *p* < 0.01.

Purified rSmAP displays clear enzymatic activity; in Figure 2A, the ability of 0.2 µg rSmAP to cleave the artificial substrate *p*-NPP over time is illustrated (the chemical structure of *p*-NPP is shown). It is clear that live schistosomula exhibit the same activity as do, to a greater degree, homogenates of schistosomula (Figure 2A, compare “Schistosomula lysate” versus “Live schistosomula”). Medium in which the parasites were cultured shows no enzyme activity (Figure 2A, “Medium control”).

Figure 2B shows that individual live adult parasites also cleave *p-*NPP and lysates of individuals exhibit significantly greater substrate cleavage. This is the case for both male (blue) and female adult worms (red), *p* < 0.01. In these experiments, lysates cleave about double that of live parasites.

**Figure 3:**
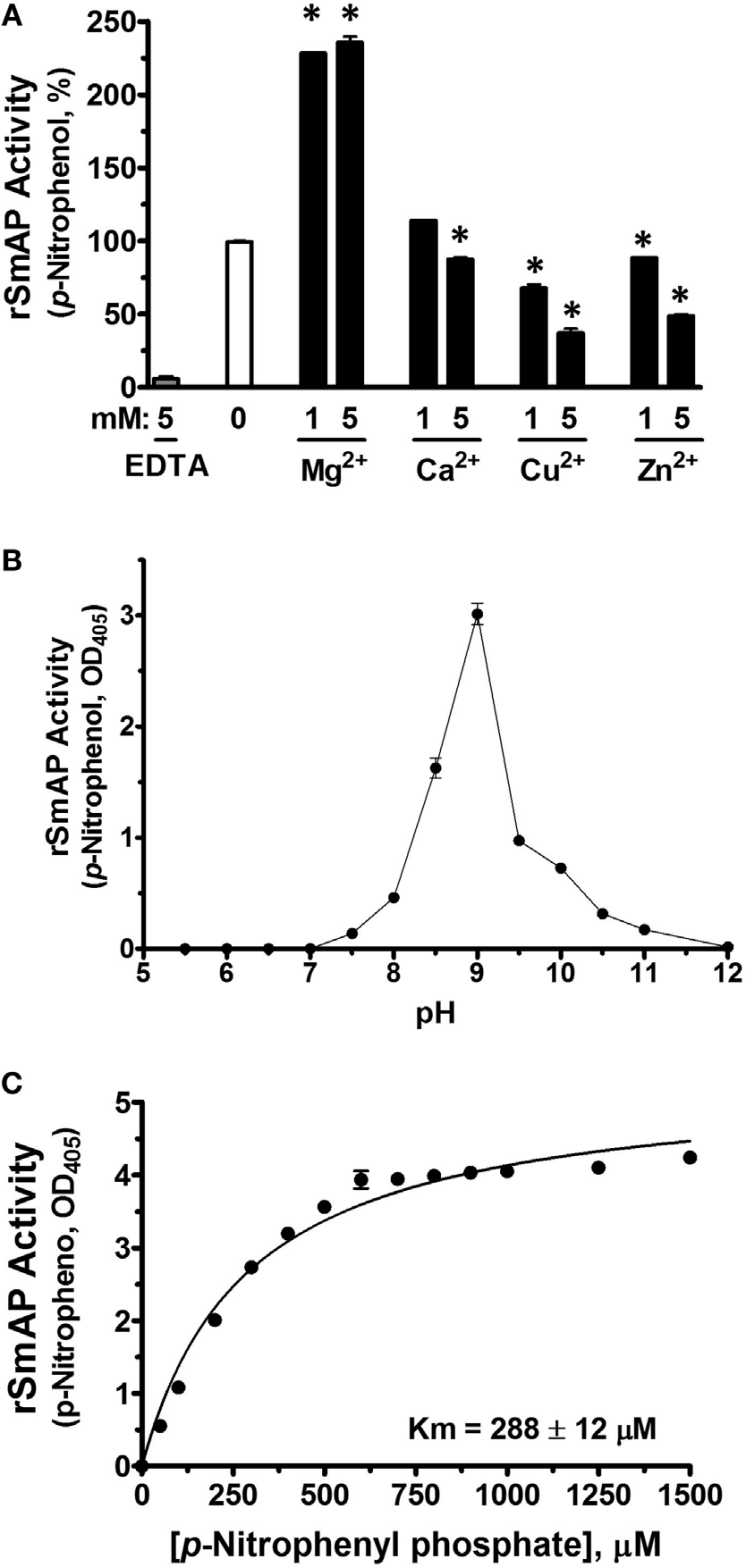
Characterization of rSmAP. **(A)** Relative activity of rSmAP (*p*-NPP cleavage, mean OD_405_ ± SD) in the presence of 0, 1, or 5 mM of added metal cations (Mg^2+^, Ca^2+^, Cu^2+^, or Zn^2+^) or 5 mM ethylenediaminetetraacetic acid (EDTA), as indicated. Activity of rSmAP in buffer lacking added metal cations (0) is set at 100%. * indicates statistically significant difference compared to no additive (0; *t*-test, *p* < 0.05). **(B)** pH preference of rSmAP in the hydrolysis of *p*-NPP. **(C)** Michaelis–Menton plot of SmAP-mediated *p*-NPP cleavage kinetics; the Km of rSmAP for *p*-NPP is 288 ± 12 µM, derived from three independent experiments.

As shown in Figure 3A, adding Mg^2+^ (at 1 or 5 mM) to the assay buffer significantly enhances rSmAP activity. In contrast, other divalent cations (Ca^2+^, Zn^2+^, or Cu^2+^) do not promote rSmAP activity; indeed, at 5 mM, all of these significantly impair enzyme activity (*p* < 0.05). Removing cations from the reaction buffer by adding the divalent ion chelator EDTA (5 mM) eliminates rSmAP activity (Figure 3A, EDTA). The pH preference of rSmAP for *p*-NPP cleavage was assessed in the range of 5.5–12 and Figure 3B reveals that the enzyme exhibits optimal activity at pH 9. Figure 3C is a Michaelis–Menton plot, illustrating the kinetics of rSmAP-mediated *p*-NPP cleavage; the Km of rSmAP for *p*-NPP is 288 ± 12 µM.

**Figure 4:**
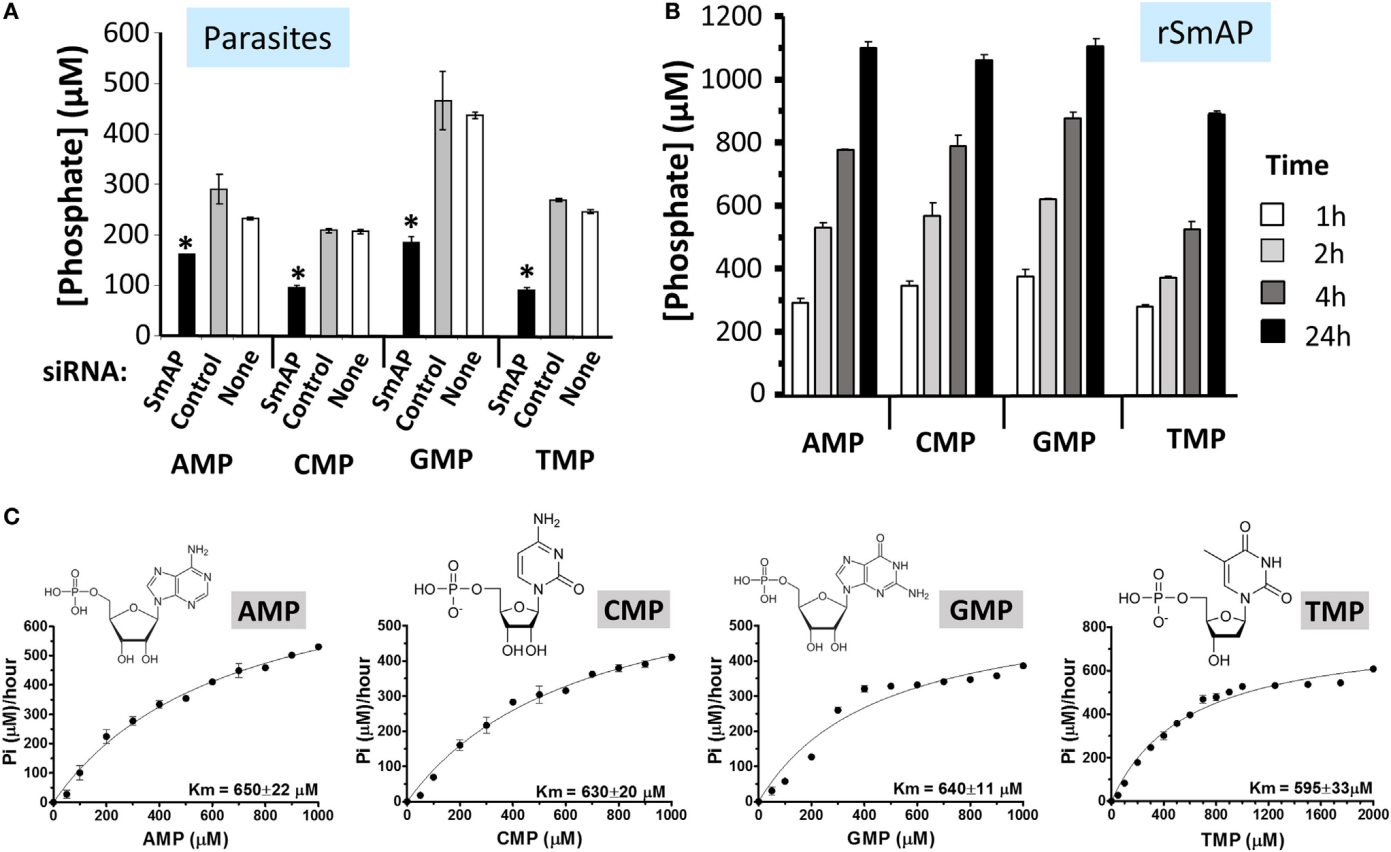
**(A)** *Schistosoma mansoni* alkaline phosphatase (SmAP) gene suppression by RNAi. Phosphate generation (μM, mean ± SD) by live schistosomula (groups of 1,000) 7 days after treatment with SmAP siRNA or an irrelevant siRNA (Control) or no siRNA (None) in the presence of different nucleoside monophosphates (NMPs) (AMP, CMP, GMP, or TMP, as indicated). In each case, parasites treated with SmAP siRNA generate significantly less phosphate compared to controls (*, one-way ANOVA, *p* < 0.05). **(B)** Phosphate generation (μM, mean ± SD) by rSmAP over time with AMP, CMP, GMP, or TMP (as indicated) as substrate. **(C)** Michaelis–Menton plots of SmAP-mediated AMP, CMP, GMP, or TMP (as indicated) cleavage kinetics, generated, and analyzed using GraphPad Prism 5.0; the chemical structures of each of the NMPs as well as the mean Km values of rSmAP for each is given. Km values were derived from three independent experiments.

Figure 4 shows data relating to the ability of SmAP to cleave four common nucleoside monophosphates (NMPs). These are: AMP, CMP, GMP, and TMP. In one experiment, living parasites were first treated with an siRNA to suppress SmAP gene expression or with a control siRNA or with no siRNA. This treatment consistently results in more than 90% suppression of SmAP RNA (16). Next, the ability of all parasites to cleave the four NMPs was assessed 7 days post siRNA treatment. Figure 4A shows that living parasites that have had their SmAP gene suppressed are significantly impaired in their ability to cleave these NMPs compared to controls (*p* < 0.05, in all cases). Figure 4B shows that rSmAP alone can cleave AMP, CMP, GMP, and TMP; in each case, increasing amounts of cleaved phosphate are detected over time. Figure 4C gives the chemical structures of each NMP as well as Michaelis–Menton plots illustrating the similar Km values of rSmAP for these NMPs, which range from 595 µM (for TMP) to 650 µM (for AMP).

### Schistosomes Cleave S1P

**Figure 5:**
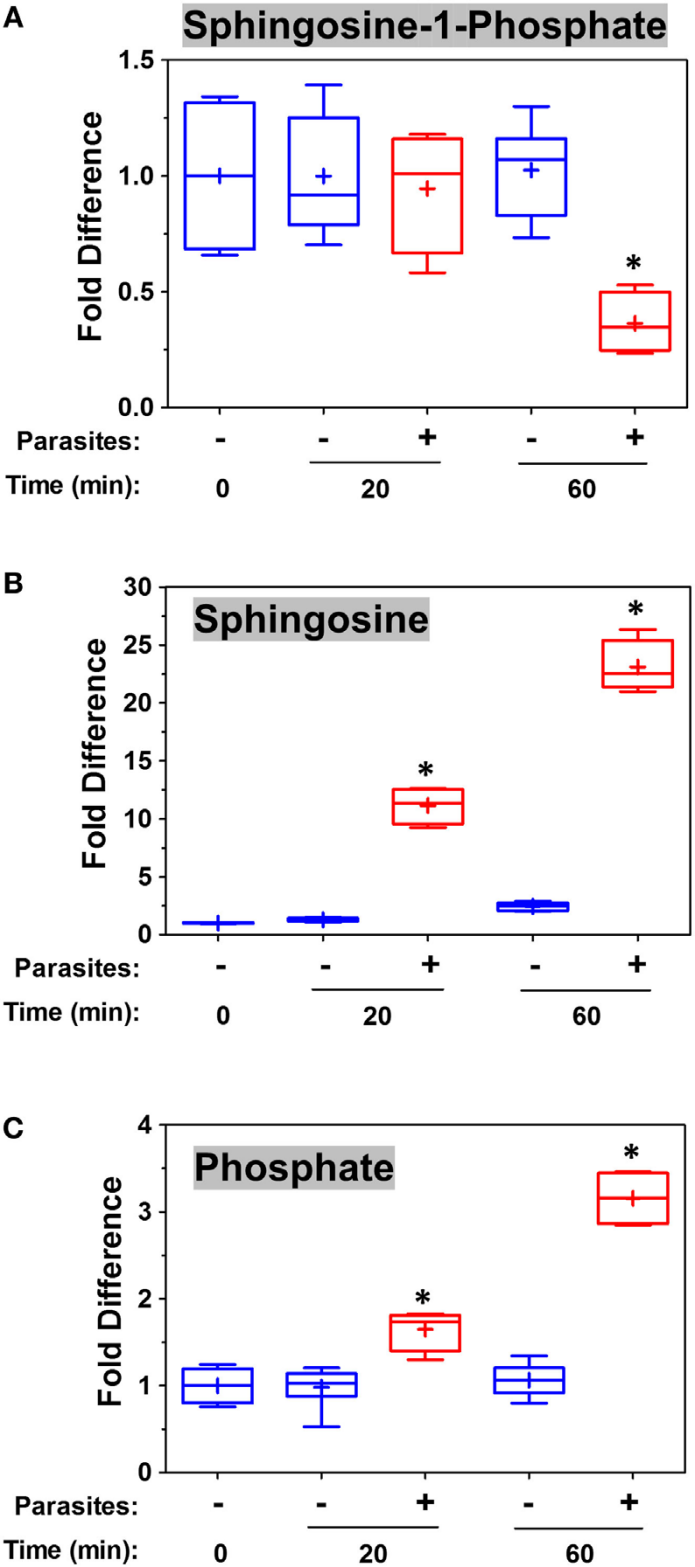
Box plots showing relative differences in the levels of Sphingosine-1-Phosphate [**(A)**, top], Sphingosine [**(B)**, middle], and Phosphate [**(C)**, bottom] in murine plasma that either contained adult schistosomes (**+**, red) or did not contain schistosomes (−, blue) for the indicated time periods. * indicates statistically significant difference compared to the same time point lacking parasites; Welch’s two-sample *t*-test, *p* < 0.05. Each box bounds the upper and lower quartile, the line in each box is the median value and “**+**” signifies the mean value for the sample; error bars indicate the maximum (upper) and minimum (lower) distribution. Values obtained at zero time (0) are set at 1.

**Figure 6:**
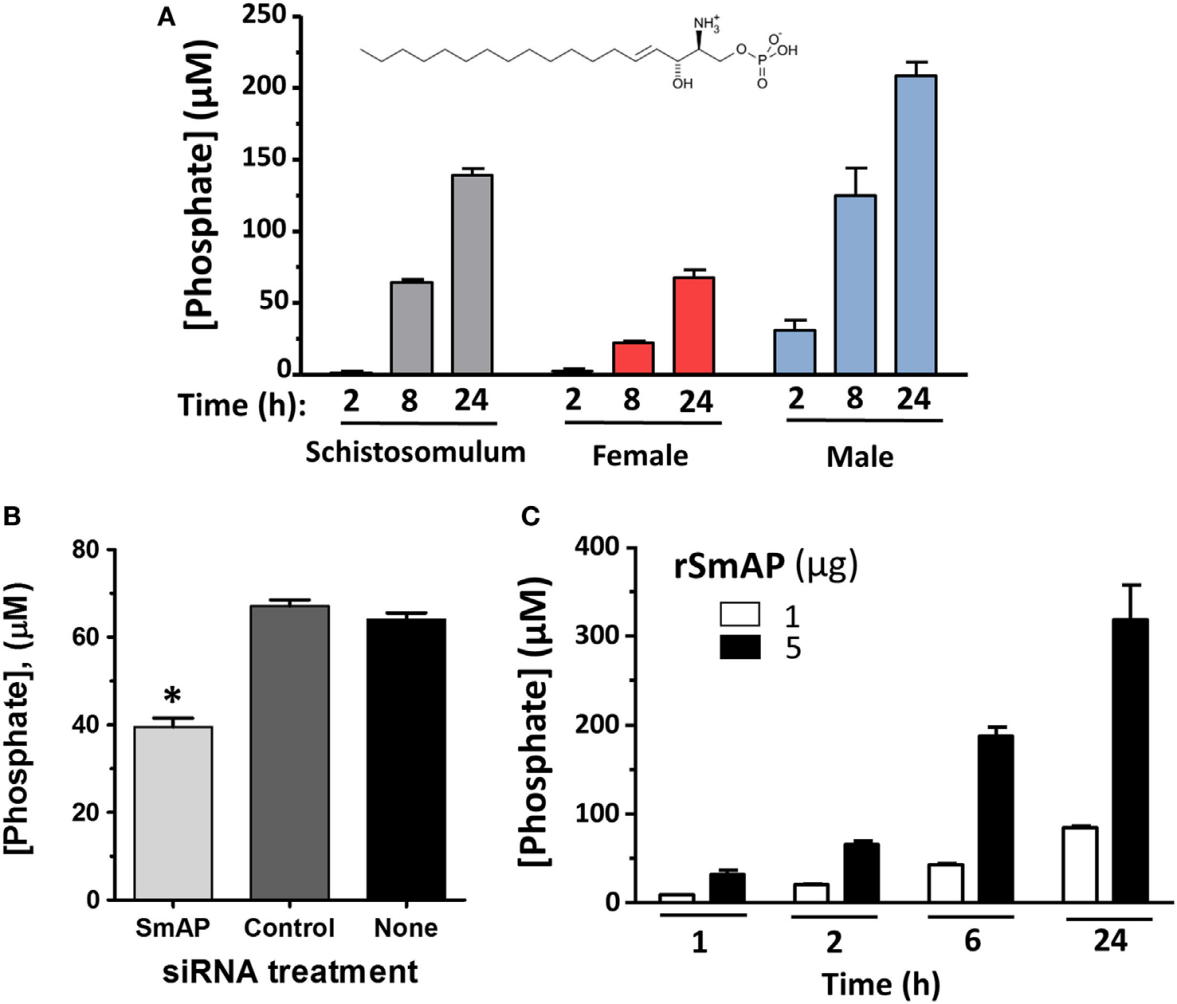
**(A)** Phosphate generation (μM, mean ± SD) by live schistosomula (groups of 1,000, gray bars) or individual adult females (red bars) or individual adult males (blue bars) in the presence of sphingosine-1-phosphate (S1P). The chemical structure of S1P is shown. **(B)** Phosphate generation (μM, mean ± SD) by individual live adult male worms 7 days after treatment with *Schistosoma mansoni* alkaline phosphatase (SmAP) siRNA or an irrelevant siRNA (Control) or no siRNA (None) in the presence of S1P. Parasites treated with SmAP siRNA generate significantly less phosphate compared to controls (*, one-way ANOVA, *p* < 0.001). **(C)** Phosphate generation (μM, mean ± SD) by rSmAP (1 or 5 µg) over time with S1P as substrate.

To examine changes brought about by schistosomes on host plasma, adult worms were first incubated in murine plasma as described in Section “Materials and Methods.” 20 and 60 min later, samples were collected and changes to the plasma metabolome were measured. Figure 5 illustrates relative changes in three metabolites [S1P (top panel), sphingosine (middle panel), and phosphate (lower panel)] from murine plasma, which contained (+) or did not contain (−) adult schistosomes. Figure 5A shows that there is a significant drop in the level of S1P at the 60-min time point in the plasma sample that contained worms, and this is accompanied by a significant increase in plasma sphingosine (shown in Figure 5B) as well as a significant increase in free phosphate detected, as shown in Figure 5C (*p* < 0.05 in each case). Since these data suggest that the worms can cleave S1P to generate sphingosine and phosphate, we next examined the ability of the worms to hydrolyze commercially obtained S1P. Figure 6A shows that all intravascular schistosome life stages [schistosomula (gray bars in Figure 6A), adult females (red bars), and adult males (blue bars)] can indeed cleave S1P to release phosphate (which accumulates over time). The chemical structure of S1P is given. Next, we set out to test the hypothesis that tegumental SmAP is responsible for the S1P cleavage seen. Adult worms were first treated with siRNAs targeting SmAP or with control siRNAs or with no siRNA and the ability of all worms to hydrolyze S1P was compared 7 days later. Figure 6B shows the results of this experiment, and it is clear that worms whose SmAP gene has been suppressed cleave significantly less S1P compared to control worms treated either with the control siRNA or with no siRNA (*p* < 0.001). Finally, as shown in Figure 6C, rSmAP, tested at two different concentrations (1 and 5 μg/reaction) cleaves S1P.

## Discussion

In this work, rSmAP was generated and purified using a CHO cell expression system. The recombinant protein is of high purity as assessed by SDS-PAGE and Coomassie staining, and it is recognized by commercially generated anti-SmAP antibodies (as well as antibodies directed to the recombinant protein’s myc tag). Analysis of rSmAP by SDS-PAGE shows it to run at a slightly higher molecular weight protein than predicted (~60 versus 57 kDa). The difference may reflect glycosylation of the recombinant protein. Indeed, treatment of rSmAP with, PNGase F (an *N*-glycan cleaving enzyme), results in a small but discernable protein mobility shift following SDS-PAGE. Similarly, treatment of the native protein in worm extracts also leads to a mobility shift showing that SmAP is a glycosylated protein (17). This result is in agreement with earlier work in which the enzyme was purified from extracts of adult worms by lectin (Concanavalin A) agarose affinity chromatography (40). SmAP protein sequence analysis shows it to possess several potential *N*-linked glycosylation sites (16).

Here, we show that rSmAP can hydrolyze the artificial substrate *p*-NPP in a reaction that requires Mg^2+^ ions. Other cations (Ca^2+^, Cu^2+^, Zn^2+^) cannot substitute for Mg^2+^ and taking away cations by treating the protein extract with the chelator EDTA abolishes enzyme activity. These data are consistent with an earlier report in which the alkaline phosphatase activity of isolated *S. mansoni* tegumental material was stimulated by 10 mM Mg^2+^ but inhibited by >1 mM Ca^2+^ (41). Adding increasing amounts of non-Mg^2+^ cations to the reaction measured here diminishes enzyme activity, presumably by competing for efficient Mg^2+^ binding to the protein.

Like other schistosome ectoenzymes that are well expressed at the host–parasite interface [e.g., the nucleotide pyrophosphatase/phosphodiesterase SmNPP5 and the ATP diphosphohydrolase SmATPDase1 (12, 16, 41)], SmAP too displays highest activity under alkaline conditions. The enzyme’s pH optimum is 9. How enzymatically efficient alkaline-loving parasite ectoenzymes like SmAP are in the host vasculature is unclear given that blood, the habitat of the worms studied here, is strongly buffered and maintains a neutral pH. Additionally, the adult worms excrete large amounts of lactate (42, 43), which would serve to acidify the parasite’s local environment, potentially further dampening the activity of these tegumental ectoenzymes.

Early work revealed alkaline phosphatase activity in the parasite surface membranes (44, 45) and, almost certainly, the activity of SmAP was being measured. More recent tegument proteomic studies (46–48), as well as immunolocalization experiments (16, 17), confirm that SmAP is found in the parasite surface membranes. We show here that living schistosomes (schistosomula as well as adult male and female worms) can cleave the alkaline phosphatase substrate *p*-NPP. We have previously shown that live schistosomula whose SmAP gene is suppressed by RNAi are severely diminished in their ability to cleave this substrate (16). In this work, we provide direct evidence that rSmAP can cleave *p*-NPP.

Immunolocalization data reveal that SmAP, in addition to being in the tegument, is also found in the parasite’s internal tissues (16). This is consistent with our finding that parasite lysates (schistosomula as well as adult male and female worms) display greater *p*-NPP cleaving ability compared to the live worms. Activity displayed by living parasites represents the action of SmAP enzyme that is located at the host–parasite interface whereas lysates contain both surface and internal SmAP. The activity measured for individual males versus individual females is comparable. So, while individual females are considerably smaller than their male counterparts, they do display higher relative expression of the SmAP gene (16). Earlier work examining isolated adult tegumental material reported that >75% of total alkaline phosphatase activity was found in these “epidermis membranes” (49). Our experiments involving live worm alkaline phosphatase activity suggest that tegumental activity accounts for somewhat less (~50%) of that measured in total parasite lysates. This is true for schistosomula as well as adult males and females. Note that medium in which schistosomula were cultured for 24 h does not have SmAP activity showing that the protein has not been excreted or secreted from the parasites to any measurable extent during culture.

Living schistosomes can also cleave several NMPs: AMP, CMP, GMP, and TMP. We show here that parasites whose SmAP gene has been suppressed are substantially diminished in their ability to cleave these substrates. rSmAP also cleaves these NMPs and with a generally similar Km (~600–650 μM). SmAP-mediated cleavage of AMP leads to the generation of adenosine (16) which, by signaling through purinergic receptors, could dampen host immune responses (7, 50) as well as inhibit platelet aggregation and block thrombus growth (21, 51). Of course, adenosine generated by the SmAP-mediated dephosphorylation of AMP may additionally (or instead) be taken up by the parasites as a nutrient (16). Since schistosomes cannot make purines *de novo* (52), salvage of such metabolites from the host is vital. In a similar manner, SmAP-mediated dephosphorylation of CMP, GMP, and TMP may generate the nutrients cytosine, guanine, and thymine in the immediate environment of the parasites from where these might be conveniently and efficiently taken in.

Clues as to other biomolecules that might act as substrates for SmAP arose following our analysis of the metabolome of murine plasma in which adult schistosomes were incubated for 1 h. Such a plasma sample, compared to a control, shows a diminution in S1P levels along with a concomitant increase in sphingosine and phosphate. This suggests that schistosomes can cleave S1P to generate its component parts. The fold increase in sphingosine and phosphate is notably greater than the fold decrease in S1P, suggesting that sources other than S1P account for much of the accumulated sphingosine and phosphate. To test the hypothesis that schistosomes can indeed hydrolyze S1P (and not merely recruit or activate a host enzyme with this function), living intravascular life-stage parasites (schistosomula and adult males and females) were each incubated with commercially obtained S1P. At selected times thereafter, S1P cleavage was examined by monitoring the level of phosphate released. In each case, this was observed. Furthermore, parasites whose SmAP gene was suppressed using RNAi were significantly impaired in their ability to cleave S1P. Consistent with this finding is the observation that rSmAP can, itself, cleave S1P to liberate phosphate. This is the first report of any parasite possessing the ability to cleave this important bioreactive metabolite.

Sphingosine-1-phosphate is a lipid signaling molecule that plays a key role in the orchestration of immune responses. It binds to a collection of G-protein-coupled receptors (S1P receptors 1 to 5) leading to downstream cellular and signaling effects. S1P is enriched in blood and lymph, whereas it is found at much lower levels in the interstitial fluids of tissues, creating a steep S1P gradient (53) that is used to control the trafficking of immune cells like lymphocytes, dendritic cells, and neutrophils (54). S1P can be secreted by monocytes and vascular endothelial cells (22, 23) and during some inflammatory reactions, “a burst of S1P” is reported to become available to its receptors in the extravascular compartment likely leading to tissue responses (53). Elevated local S1P concentrations have been postulated to play an important role in guiding immune cells to sites of local injury (33). Processes like lymphocyte and innate lymphoid cell circulation, leukocyte recruitment and positioning, antigen presentation, and inflammation can all be impacted by local and systemic S1P levels and by S1P receptors on immune cells (22, 23, 32). By degrading S1P using SmAP, we hypothesize that schistosomes contain any S1P burst and dampen associated parasite-damaging host responses.

Recent studies have revealed that S1P signaling is not just involved in immune cell function but is also actively coupled with coagulation processes (33). During vascular injury, the coagulation proteases thrombin and activated factor X can enhance the synthesis and release of S1P from vascular smooth muscle cells (33). Platelets, which contain high concentrations of S1P, can release it during coagulation (55). In addition, S1P, acting on S1P receptor 1 expressed by platelets, can enable platelet aggregation in response to protease-activated receptor 4-peptide and ADP (36). For schistosomes, the action of tegumental SmAP would likely diminish the local concentration of S1P to ameliorate its downstream pro-coagulant effects. Finally, S1P has been shown to be critical for thrombopoiesis (56), and it is possible that schistosome mediated destruction of this metabolite could contribute to the decline in platelet numbers observed in infected vertebrate hosts (57).

This is the first report of an ability of any pathogen to target and degrade extracellular S1P. It has been reported that some pathogenic bacteria can subvert intracellular S1P pathways to promote survival. For instance, within macrophages, *Burkholderia pseudomallei* and *B. thailandensis* secrete S1P-cleaving enzymes (S1P lyases) that are required for replication and virulence (58). A *Legionella pneumophila* S1P lyase restrains autophagy within infected macrophages and contributes to bacterial virulence (59). In these examples, the pathogen enzymes target intracellular S1P; in the case of schistosomes, it is an ectoenzyme—tegumental SmAP—degrading extracellular S1P levels that may impede S1P signaling and promote parasite survival.

We are now developing a more complete understanding of the molecular capabilities of the intravascular *S. mansoni* tegument. The tegument is more than a surface for the uptake and exchange of metabolites (60). It contains a collection of proteins that can profoundly impact the biochemistry of the parasite’s local environment. For instance, host-interactive tegumental proteases can cleave key components of the coagulation cascade like fibronectin (61) and high molecular weight kininogen (62). Tegumental ATP diphosphohydrolase, SmATPDase1, can cleave the pro-inflammatory mediator ATP as well as the procoagulant ADP (12, 63). The ectonucleotide pyrophosphatase/phosphodiesterase SmNPP5 can additionally cleave ADP and has been shown to block platelet aggregation *in vitro* (13). In addition, the parasites express proteins at their surface (e.g., SmEnolase) that can act to recruit and enhance the activation of the thrombus-degrading enzyme plasminogen (64). The tegumental ectoenzyme under study here—SmAP—contributes to these capability’s by converting AMP to its anti-inflammatory, anti-thrombotic derivative adenosine (16) and, as shown in this report, by degrading the pro-inflammatory and pro-coagulant lipid mediator S1P. All of these molecular effects likely contribute to the known ability of living schistosomes to hamper blood coagulation *in vitro* (65), to remain overtly unmolested by immune and coagulation effectors *in vivo* (5) and to survive for many years within the vasculature of their hosts.

## Ethics Statement

All protocols involving animals were approved by the Institutional Animal Care and Use Committees (IACUC) of Tufts University under protocol G2015-113. All experimental procedures were carried out in accordance with approved guidelines of the IACUC.

## Author Contributions

ME, AD, and PS: experiment design. ME, AD, RB, and QW: experiment performance. ME, AD, RB, and PS: data analysis. ME, AD, MA, E-SE-K, SE-B, and PS: manuscript preparation.

## Conflict of Interest Statement

The authors declare that the research was conducted in the absence of any commercial or financial relationships that could be construed as a potential conflict of interest.
